# *Bacteroides thetaiotaomicron* generates diverse α-mannosidase activities through subtle evolution of a distal substrate-binding motif

**DOI:** 10.1107/S2059798318002942

**Published:** 2018-04-24

**Authors:** Andrew J. Thompson, Richard J. Spears, Yanping Zhu, Michael D. L. Suits, Spencer J. Williams, Harry J. Gilbert, Gideon J. Davies

**Affiliations:** a Department of Chemistry, University of York, Heslington, York YO10 5DD, England; b Institute for Cell and Molecular Biosciences, Newcastle University, Framlington Place, Newcastle-upon-Tyne NE2 4HH, England; c School of Chemistry and Bio21 Molecular Science and Biotechnology Institute, University of Melbourne, Parkville, Victoria, Australia

**Keywords:** glycans, carbohydrates, α-mannosidase, substrate specificity, *Bacteroides thetaiotaomicron*, glycoside hydrolase family 92

## Abstract

A dominant human gut microbe, the well studied symbiont *Bacteroides thetaiotaomicron* (*Bt*), is a glyco-specialist that harbors a large repertoire of genes devoted to carbohydrate processing. Despite strong similarities among them, many of the encoded enzymes have evolved distinct substrate specificities, and through the clustering of cognate genes within operons termed polysaccharide-utilization loci (PULs) enable the fulfilment of complex biological roles. Structural analyses of two glycoside hydrolase family 92 α-mannosidases, BT3130 and BT3965, together with mechanistically relevant complexes at 1.8–2.5 Å resolution reveal conservation of the global enzyme fold and core catalytic apparatus despite different linkage specificities. Structure comparison shows that *Bt* differentiates the activity of these enzymes through evolution of a highly variable substrate-binding region immediately adjacent to the active site. These observations unveil a genetic/biochemical mechanism through which polysaccharide-processing bacteria can evolve new and specific biochemical activities from otherwise highly similar gene products.

## Abstract 2

Analysing two sequence-related bacterial glycoside hydrolase family 92 mannosidases with distinct functions, a structural basis for their varied specificities is revealed.

## 1. Introduction   

Prokaryote-encoded α-mannosidase enzymes, particularly those deployed by the human gut microbiota, have garnered significant interest in recent years owing to their importance in digestive health. Furthermore, these highly accessible model proteins share significant structural and mechanistic similarity with complex mammalian homologues, and have provided new insights into the intricate nucleophilic substitution reactions that are the hallmark of mannosidase chemistry (Suits *et al.*, 2010 ▸; Thompson *et al.*, 2012 ▸; Offen *et al.*, 2009 ▸; Tailford *et al.*, 2008 ▸). Emerging research has revealed that certain symbiotic gut-resident bacteria maintain a selective advantage over microbial competitors through the ability to catabolize α-linked mannopolysaccharides as a sole carbon source (Cuskin *et al.*, 2015 ▸). One such highly prevalent bacterium, *Bacteroides thetaiotaomicron* (*Bt*), encodes large numbers of enzymes from α-mannoside-specific glycoside hydrolase (GH) families, including GH38, GH76, GH92 and GH125 [family definitions according to the sequence-based CAZy classification system (Lombard *et al.*, 2014 ▸); see http://www.cazy.org and http://www.cazypedia.org]. Within the *Bt* genome, enzymes from these families are organized and regulated in a highly systematic fashion within polysaccharide-utilization loci (PULs; see Martens *et al.*, 2009 ▸), which allows the rapid and simultaneous expression of a complete molecular ‘toolkit’ to catalyse the breakdown of otherwise inaccessible high-mannose dietary components such as yeast α-mannan and mammalian high-mannose N-glycans. Unlike many other carbohydrate-hydrolysing pathways, which allow shared access to metabolic byproducts in a symbiotic relationship with the host, *Bt* uniquely employs mannan/mannoside-targeted PULs in a purely ‘selfish’ mechanism (Cuskin *et al.*, 2015 ▸), emphasizing a selectively advantageous role for these gene/enzyme catalogues compared with fellow gut-resident microbial species. As such, the biochemical properties and correct regulation of enzymes within these loci is crucial in the maintenance of a balanced and diverse gut microbial environ­ment and in the overall healthy functioning of the microbiota.

Most enzymes within family GH92 are exo-acting α-mannosidases that are capable of hydrolysing terminal mannosidic residues with a variety of linkages, including α-1,2, α-1,3, α-1,4 and α-1,6 glycosidic bonds (Zhu *et al.*, 2010 ▸; Robb *et al.*, 2017 ▸). One enzyme has been shown to possess mannan-specific ‘decapping’ activity, hydrolysing mannose from Man-α-1,6-PO_4_-Man linkages (Tiels *et al.*, 2012 ▸). To date, the structures of two *Bt* GH92 α-mannosidases, BT3990 and BT2199, have been reported, which are both α-1,2-mannosid­ases. These enzymes comprise a large two-domain structure, with a centrally positioned active centre formed from elements of both the major N- and C-terminal domains (Zhu *et al.*, 2010 ▸). Complementary to α-mannosidases within largely eukaryotic mannosidase families (particularly GH38 and GH47) that play important roles in N-glycan processing, GH92 enzymes are metal-dependent, featuring a single Ca^2+^ ion coordinated within the catalytic active site. For each of these families, divalent metal ions assist in both binding and distortion of the substrate away from the characteristic ^4^ *C*_1_ ground-state conformation, thus lowering the large energy barrier associated with nucleophilic attack at the anomeric centre of α-mannosides (Vallée *et al.*, 2000 ▸; Numao *et al.*, 2003 ▸; Karaveg *et al.*, 2005 ▸; Thompson *et al.*, 2012 ▸). GH92 enzymes act *via* a classic single-displacement mechanism, leading to inverted configuration at the anomeric centre (Zhu *et al.*, 2010 ▸). To date, 22 GH92 enzymes from *Bt* have been biochemically characterized. Six enzymes have specific activity against α-1,2-linked mannosides, six target α-1,3 linkages, four target α-1,4 linkages and one (BT3994) exhibits mixed activity against α-1,3-, α-1,4- and α-1,6-mannosides (Zhu *et al.*, 2010 ▸). Further substrate diversity is seen for the GH92 family member CcMan5 from *Cellulosimicrobium cellulans*, which targets Man-α-1,6-PO_4_-Man phosphodiester linkages (Tiels *et al.*, 2012 ▸). The specificity of a further five *Bt* enzymes has not yet been identified through screening against simple di­saccharides, although each has been shown to degrade both mammalian high-mannose-type N-glycans and *Saccharomyces cerevisiae* cell-wall α-mannan, potentially indicating more complex substrate specificities (Zhu *et al.*, 2010 ▸).

Structural analysis of *Bt* GH92 enzymes to date has been confined to two similar α-1,2-specific enzymes; we now seek to understand the underlying structural basis for the variety of activities displayed within this group. Here, we present the structures of two additional *Bt* GH92 α-mannosidases, BT3130 and BT3965, as well as of mechanistically relevant complexes. We show that despite alternate linkage preferences, ligand complexes with both BT3130 (linkage specificity uncertain; likely α-1,3-specific) and BT3965 (α-1,4-mannosidase) indicate significant conservation in both reaction mechanism and conformation at the transition state (TS^‡^) right across the GH92 family. Varying structural features located within a subsite immediately external to the catalytic active site, and likely contributing to the variable substrate specificities displayed by *Bt* GH92 enzymes, are identified and discussed.

## 2. Materials and methods   

### 2.1. Protein production and purification   

The genes encoding BT3130 and BT3965 were cloned exactly as described previously in Zhu *et al.* (2010 ▸). Briefly, the respective enzyme-encoding genes were amplified from a *Bt* genomic DNA template using the primers shown in Table 1 ▸. BT3130 was modified to remove a predicted signal peptide (*SignalP*; Nielsen, 2017 ▸) formed by the 18 N-terminal amino acids. Amplified products for both genes were digested with NcoI and XhoI restriction endonucleases prior to ligation into a pre-treated pET-21a *Escherichia coli* expression vector. The final pET-21a-BT3130 and pET-21a-BT3965 expression constructs comprised native codons 19–733 and 2–756, respectively, encoding a C-terminal His_6_ purification tag (see Table 1 ▸). Gene expression and protein purification were identical for both enzymes. *E. coli* Tuner cells harbouring either pET-21a-BT3130 or pET-21a-BT3965 were cultured at 37°C (310 K) to mid-exponential phase and were induced by the addition of 0.2 m*M* IPTG (final concentration), incubating at 16°C (289 K) overnight. The cell pellets were lysed in 50 m*M* HEPES pH 7.0, 300 m*M* NaCl, 2 m*M* imidazole and the proteins were purified by Ni–NTA affinity chromatography, eluting *via* gradient exchange into the same buffer containing 500 m*M* imidazole. Finally, the protein samples were loaded onto a Superdex 200 (16/60) size-exclusion column equilibrated with 50 m*M* HEPES pH 7.0, 300 m*M* NaCl. Individual peak fractions were collected and concentrated to between 23.8 and 56.0 mg ml^−1^ for various samples.

### 2.2. Crystallization   

Both BT3130 and BT3965 were screened for crystallization hits against a variety of commercially available sparse-matrix screens, including Crystal Screen, Crystal Screen 2 and Index from Hampton Research, and PACT 1 and 2, JCSG-*plus*, The PGA Screen and Morpheus from Molecular Dimensions. All screens were conducted as sitting-drop experiments in standard two-drop, 96-well MRC plates using both 1:1 and 1.5:1 protein:reservoir ratios and were incubated at 19°C. Screening drops for both BT3130 (stock at 23.8 mg ml^−1^) and BT3965 (stock at 56.0 mg ml^−1^) were prepared by mixing 150 nl protein solution with 150 nl reservoir solution (a 1:1 ratio) and by mixing 150 nl protein solution with 100 nl reservoir solution (a 1.5:1 ratio). Final crystals of BT3130 suitable for data collection and structure solution were grown at 19°C using hanging-drop vapour diffusion (details are given in Table 2 ▸). Pure BT3130 (23.8 mg ml^−1^) was mixed in a 2:1 ratio with a reservoir solution consisting of 18%(*w*/*v*) PEG 3350, 0.1 *M* bis-tris propane pH 6.4, 0.2 *M* sodium bromide. Diffraction-quality BT3965 crystals were grown under identical conditions using a 2:1 mixture of pure protein solution (56.0 mg ml^−1^) and reservoir solution consisting of 20%(*w*/*v*) PEG 3350, 0.2 *M* sodium nitrate. Complexes of both enzymes with the α-mannosidase inhibitor mannoimidazole (ManI; Granier *et al.*, 1997 ▸) were obtained by soaking native crystals in 10 m*M* ManI (final concentration) for a period of 30 min. All crystals were cryoprotected prior to flash-cooling in liquid nitrogen *via* the addition of 30%(*w*/*v*) ethylene glycol (final concentration) with or without exogenous Ca^2+^ (1 m*M* final concentration).

### 2.3. Data collection and processing   

Diffraction data for native BT3130 and for the complex with ManI (Table 3 ▸) were collected on beamlines I04-1 and I03, respectively, at Diamond Light Source (DLS), Didcot, England. Diffraction data for native BT3965 and for the ManI complex (Table 4 ▸) were collected on DLS beamlines I04 and I04-1, respectively. All diffraction data were processed using a combination of *XDS* (Kabsch, 2010 ▸) and *AIMLESS* (Evans & Murshudov, 2013 ▸) within the *CCP*4 program suite (Winn *et al.*, 2011 ▸).

### 2.4. Structure solution and refinement   

The structures of native BT3130 and BT3965 were solved by molecular replacement employing the *CCP*4 implementation of *Phaser* (McCoy *et al.*, 2007 ▸) with the coordinates of a previously solved *Bt* GH92 enzyme, BT3990 (PDB entry 2wvy; Zhu *et al.*, 2010 ▸), as a phasing model. Initial atomic models of both enzymes were constructed using *Buccaneer* (Cowtan, 2006 ▸) and were extended by iterative rounds of manual model building and refinement using *Coot* (Emsley *et al.*, 2010 ▸) and *REFMAC* (Murshudov *et al.*, 2011 ▸). For the ligand-bound structures, the final atomic models of the respective native enzymes were refined against ligand-complex data sets, and the resulting electron-density maps were inspected visually for evidence of ligand binding. All resultant models were refined to convergence *via* the maximum-likelihood method using *REFMAC* (see Tables 5 ▸ and 6 ▸). The structures were validated using *Clipper* and *EDSTATS* within the *CCP*4 program suite (Winn *et al.*, 2011 ▸). Structure factors and final atomic models have been deposited in the PDB with accession codes 6f8z (BT3130), 6f90 (BT3130–ManI), 6f91 (BT3965) and 6f92 (BT3965–ManI). All structural figures were prepared using *CCP*4*mg* (McNicholas *et al.*, 2011 ▸).

## 3. Results and discussion   

**Figure 1:**
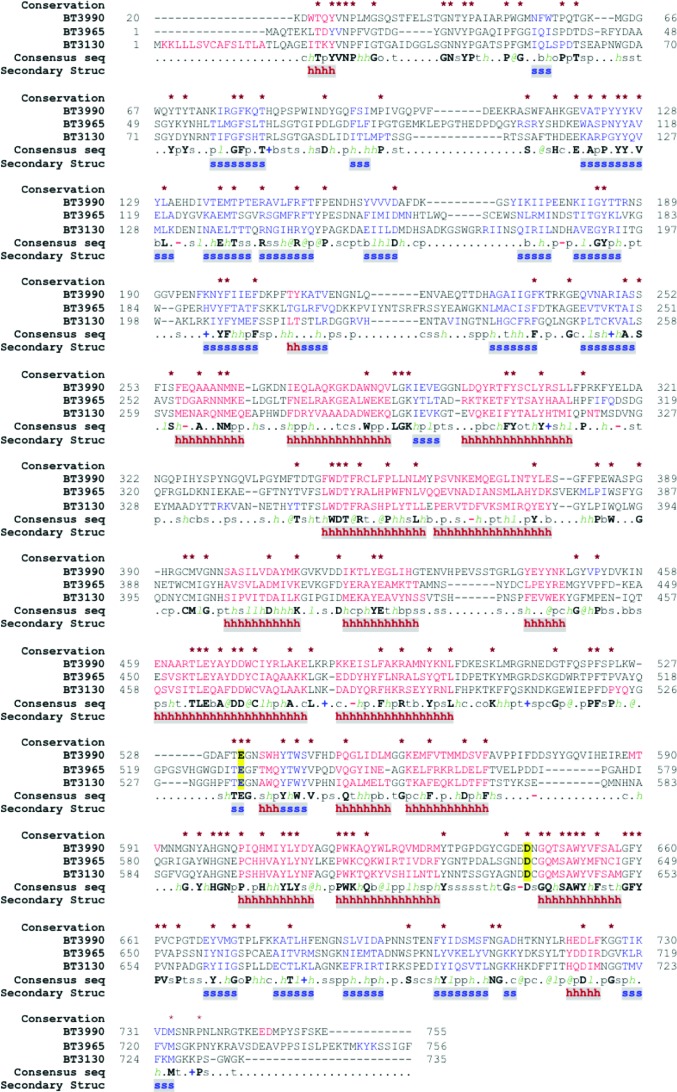
Sequence alignment of BT3990, BT3965 and BT3130. Primary-sequence and structural alignments were performed using the *PROMALS*3*D* server (Pei *et al.*, 2008 ▸). Conserved residues are indicated by asterisks above the alignment, α-­helices are shown in red and β-­strands are shown in blue. Consensus residues are indicated below the alignment: conserved amino acids are in bold uppercase letters, aliphatic residues (I, V, L) are labelled *l*, aromatic residues (Y, H, W, F) are labelled @, hydrophobic residues (W, F, Y, M, L, I, V, A, C, T, H) are labelled *h*, alcohol residues (S, T) are labelled o, polar residues (D, E, H, K, N, Q, R, S, T) are labelled p, tiny residues (A, G, C, S) are labelled t, small residues (A, G, C, S, V, N, D, T, P) are labelled s, bulky residues (E, F, I, K, L, M, Q, R, W, Y) are labelled b, positively charged residues (K, R, H) are labelled **+**, negatively charged resdidues (D, E) are labelled **−** and charged residues (D, E, K, R, H) are labelled c. Consensus secondary-structure motifs are shown on the lower line: a bold red h indicates α-helix and a bold blue s indicates β-­strand. Catalytic acid and base residues are shown in bold on a yellow background.

The three-dimensional structures of BT3130 and BT3965 both reveal a two-domain enzyme that appears to be well conserved with published examples of GH92 α-mannosidases. These enzymes feature a pocket-like central cavity composed of structural elements from both the N-terminal and C-terminal domains (Zhu *et al.*, 2010 ▸; Robb *et al.*, 2017 ▸). Kinetic analysis of site-specific enzyme variants, together with ligand-complex structures, have shown that this pocket-like region comprises the catalytic active site (Zhu *et al.*, 2010 ▸). Primary-sequence analysis and direct comparisons with available structures demonstrate strong conservation of key amino-acid side chains within this region of both BT3130 and BT3965, suggesting that the observed differences in substrate specificity are likely to be conferred by minor, local structural differences, while both the global fold and the overall reaction mechanism for these diverse α-mannosidases are broadly maintained (see Figs. 1 ▸ and 2 ▸). Indeed, quantitative structural comparison using the *DALI* server (Holm & Rosenström, 2010 ▸) shows that the three-dimensional structures of BT3130 and BT3965 have high similarity, with an r.m.s.d. of 1.4 Å mapped over 738 matched C^α^ positions (sequence identity = 40%, *Z*-score = 48.7).

**Figure 2:**
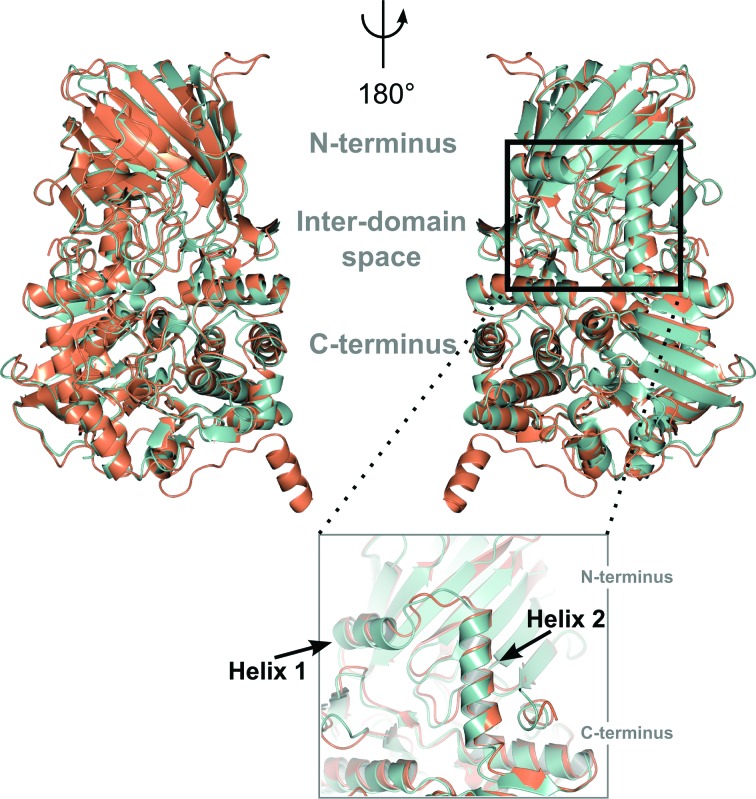
Tertiary-structure overlay of BT3130 (green) and BT3965 (orange) and (inset) a close-up view of the rear face of the molecule showing helices 1 and 2 that form the structural spine linking the N- and C-terminal domains. The left and right panels represent a 180° rotation around the vertical axis; The N-terminus, C-terminus and inter-domain space containing the catalytic active site are indicated.

The major part of the BT3130/BT3965 N-terminal domain presents as a β-sandwich motif comprising 16 antiparallel β-strands, and is formed by a continuous stretch of amino acids approximately from positions 55 to 258 (Figs. 1 ▸ and 2 ▸ *a*; for brevity, unless specified, residue numbers are approximated throughout to account for the small differences between the BT3130 and BT3965 numbering). This region bears some similarity to the accessory (noncatalytic) domains of other large glycosidases, particularly the GH38 family of retaining α-mannosidases (Numao *et al.*, 2003 ▸). However, in contrast to such families, the active site of GH92 enzymes is formed from shared structural elements rather than being located within a distinct domain, implying a more direct role in catalysis and substrate binding than is typically observed for similar β-sheet structures. Subsequent to the β-sandwich fold, the remainder of the N-terminal modules of BT3130 and BT3965 forms a near-continuous structural progression into the larger C-terminal domain. The C-terminus of the final β-sheet strand loops posteriorly, forming two α-helices positioned at approximately 90° to one another (Fig. 2 ▸ *b*). Helix 1, positioned directly behind the β-sandwich, runs laterally and forms the rear face of the molecule, contributing to the inter-domain interface. Helix 2, perpendicular to helix 1, projects directly down from the β-sheet motif into the C-terminal domain, and acts as a rigid backbone to anchor the two halves of the BT3130/BT3965 enzymes together (Fig. 2 ▸ *b*). Reminiscent of a classic catalytic module, the larger C-terminal portion (295–730) of GH92 mannosidases forms a decorated (α/α)_6_-barrel structure. Several short β-strand sections (residues 315–345 and 385–395) adorn the outer surface of the barrel, and together with the first barrel helix pack against the underside of the N-terminal β-sandwich to form the major domain interface and create a solvent-accessible cavity (Fig. 2 ▸ *a* and Supplementary Fig. S1). Located towards the middle of this inter-domain space, and comprising various flexible loops linking both individual helices of the (α/α)_6_-barrel and individual β-strands within the N-terminal domain, an array of highly conserved amino acids are positioned in close proximity and form the BT3130 and BT3965 catalytic active sites (Supplementary Fig. S1).

**Figure 3:**
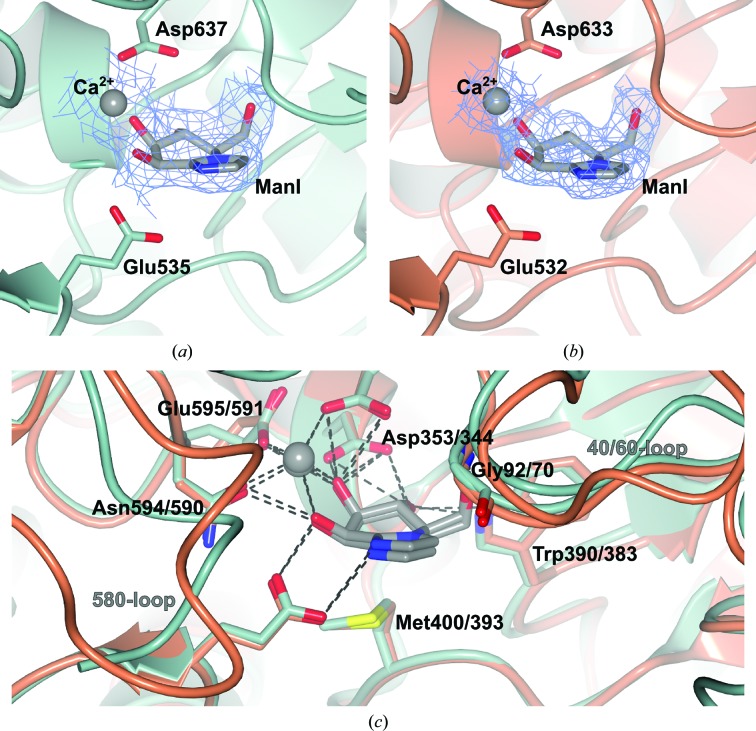
ManI and a Ca^2+^ ion bound in the catalytic active sites of BT3130 (*a*) and BT3965 (*b*). Ligands are shown together with the respective catalytic acid and base residues. The depicted electron-density maps are *REFMAC* maximum-likelihood/σ_A_-weighted 2*F*_o_ − *F*_c_ syntheses contoured at 0.15 and 0.37 e Å^−3^ (1.0σ), respectively. (*c*) Extended overlay of the BT3130 (green) and BT3965 (orange) active-site pockets. Within the −1 subsite, all side chains, structural motifs and hydrogen-bonding interactions with ManI are fully conserved (rear centre of image, black labels). Structural elements that compose the reducing-end (positive) subsites show far greater variability (front left and upper right of image, grey labels). Enzyme–ligand/ion hydrogen bonds are shown as black dashed lines.

As in the GH92 structures of BT3990 and BT2199, the locations of the active sites of both BT3130 and BT3965 were confirmed through determination of structural complexes, in this case employing the high-affinity transition-state (TS) mimic ManI (Supplementary Fig. S1 and Fig. 3 ▸). Previously, structural alignment, augmented by variant kinetic analysis, informed the assignment of catalytic and substrate-coordinating functionality to various amino-acid side chains within the active-site pocket (Zhu *et al.*, 2010 ▸). Accordingly, Glu535 and Asp637 in BT3130 (and Glu531 and Asp633 in BT3965; see Figs. 3 ▸ *a* and 3 ▸ *b*) were assigned as the respective general acid and general base residues within the catalytic mechanism. However, the conservation of homologous amino-acid residues is unrestricted to merely those directly involved in catalysis. Active-site overlays of BT3130 and BT3965, as well as family-based homology mapping of the complete enzyme monolith (Supplementary Fig. S1), demonstrate significant conservation of the catalytic pocket architecture and of many amino acids that contribute directly to either ligand binding or enzymatic function (Fig. 3 ▸ *c*). Like the members of the exo-α-mannosidase families GH38 and GH47, GH92 enzymes are metal-dependent, and a single calcium ion is observed coordinated within the active sites of both BT3130 and BT3965. Interestingly, however, while Ca^2+^ derived from the bacterial expression host was consistently observed within previous structures of BT3990, both BT3130 and BT3965 showed a somewhat reduced ability to bind and sequester this required divalent cation (see Supplementary Fig. S2). An early BT3130 structure crystallized in 200 m*M* potassium citrate showed displacement of Ca^2+^ in favour of two aberrantly coordinated K^+^ ions bound in close proximity to one another (Davies group, unpublished work). Similarly, the native structure of BT3965 shows displacement and evidence of only partial ion occupancy within the catalytic active site (see Supplementary Fig. S2*b*). It should be noted that kinetic analysis in the presence of EDTA (see Zhu *et al.*, 2010 ▸) has shown that the catalytic activity of GH92 enzymes can only be restored by the addition of Ca^2+^; likewise, crystals of BT3130 and BT3965 do not bind ManI in the absence of Ca^2+^, emphasizing the required role of calcium as both a binding partner and a catalytic cofactor. Therefore, to ensure observation of the true, catalytically competent forms of these enzymes, and the determination of meaningful ligand complexes, BT3130 was screened for crystallization with the addition of 10 m*M* calcium acetate (final concentation) to the protein buffer (in addition to avoiding K^+^-containing screen hits), while the subsequent ManI complexes of both enzymes were cryoprotected with the addition of 1 m*M* Ca^2+^ (final concentration) to the cryoprotectant solutions. Cofactor supplementation was successful, allowing determination of fully occupied enzyme–Ca^2+^–ManI complexes (Fig. 3 ▸ and Supplementary Fig. S2*c*). That the binding of required divalent ions is so poor within these enzymes, and may in fact be concomitant with substrate/ligand binding, suggests a possible route towards activity regulation, where the presence of both substrate and Ca^2+^ may be required within the same biological compartment to allow hydrolysis.

The mannosidase inhibitor ManI has been highlighted as a uniquely informative molecular probe that can be used to report on transition-state conformation for a variety of α-mannosidase families (Zhu *et al.*, 2010 ▸; Thompson *et al.*, 2012 ▸; Williams *et al.*, 2014 ▸; Tankrathok *et al.*, 2015 ▸). ManI is derived by the annulation of a deoxymannojirimycin piperidine ring with an imidazole, resulting in double-bond character between C1 and the endocyclic N atom (Terinek & Vasella, 2005 ▸), thus providing shape mimicry of the oxocarbenium ion-like character present at the TS^‡^ of glycosidase-catalysed hydrolysis. In particular, this compound has low energetic barriers between various boat and half-chair conformations, equivalent to catalytically relevant oxocarben­ium ion conformations, meaning that the observed conformation of the inhibitor ‘on-enzyme’ are those imposed by the enzyme, rather than from an intrinsic bias of the inhibitor (Williams *et al.*, 2014 ▸). ManI inhibits both BT3130 and BT3965 with approximately similar *K*_i_ values of 1.0 and 0.4 µ*M*, respectively (see the Supplementary Information to Zhu *et al.*, 2010 ▸). Complexes of ManI with BT3130 and BT3965 revealed identical distortions into approximate *B* ^‡^_2,5_ conformations, providing independent confirmation of the previously proposed ^O^ *S*_2_↔*B* ^‡^_2,5_↔^1^ *S*_5_ conformational itinerary for GH92 enzymes (Zhu *et al.*, 2010 ▸; see Fig. 3 ▸).

Characteristic of exo-active enzymes that catalyse the removal of terminal sugar moieties from the nonreducing end of substrate saccharides, ManI is located in a buried, protein-enclosed active site, defined as the −1 subsite by the GH subsite nomenclature of Davies *et al.* (1997 ▸), and makes extensive interactions with surrounding amino-acid side chains (Fig. 3 ▸ *c*). Within the active site of BT3130, ManI is coordinated within a network of interactions: hydrogen bonding of O6 to the backbone amide of Gly92 (Gly70 in BT3965), hydrogen bonding of O4 to the side-chain N atom of Trp390 and O^δ2^ of Asp353 (Trp383 and Asp344, respectively, in BT3965) and a hydrophobic stacking interaction with the side chain of Met400 (Met393 in BT3965), which is positioned directly beneath the plane of the sugar ring. The carboxylate side chain of Asp353 (Asp344 in BT3965) makes a bridging interaction, simultaneously binding to the inhibitor O3 and O4 positions. Together with dual coordination of O2 and O3 by the bound Ca^2+^ ion, these interactions position the substrate, assist in distortion away from the ground state into a pre-activated ^O^ *S*_2_ conformation for catalysis and stabilize high-energy transitions along the conformational itinerary (Fig. 3 ▸ *c*; Zhu *et al.*, 2010 ▸). Remarkably, despite differing substrate specificities (BT3130 is characterized as uncertain linkage preference, with weak activity against α-1,3-mannobiose and strong activity against yeast α-mannan, while BT3965 is a highly active α-1,4-mannosidase; Zhu *et al.*, 2010 ▸), all observed enzyme–inhibitor interactions within the −1 subsite of both proteins are fully conserved. Thus, the crucial amino acids governing mannoside-linkage preference, and therefore directing key activities within the GH92 family, are likely to be maintained external and separate to the core catalytic apparatus.

**Figure 4:**
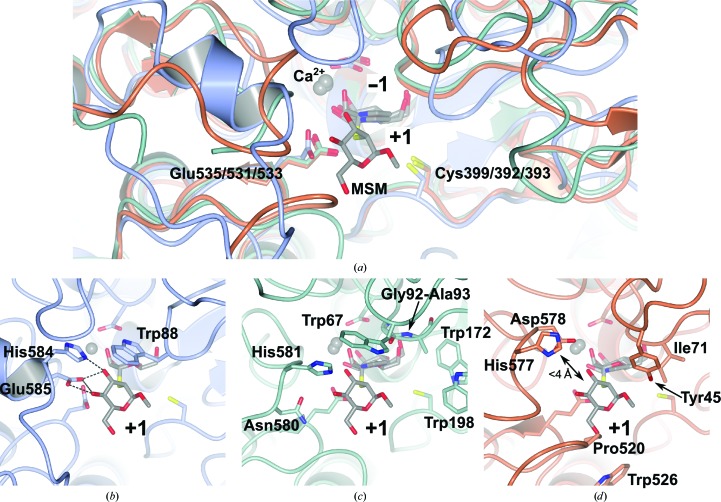
Structural superposition of *Bt* GH92 enzyme–inhibitor complexes reveals extensive diversity beyond the conserved −1 subsite. (*a*) A previously solved complex of BT3990–MSM overlaid with BT3130–ManI and BT3965–ManI. Glu535/532/533 overlays with Cys399/392/393 and the glycosidic S atom of MSM to form the subsite boundary. The positions of bound ligands, Ca^2+^ and coordinating amino acids in the −1 subsite are fully conserved. (*b*) α-1,2-Mannosidase activity in BT3990 is conferred through hydrogen bonding of the +1 mannoside to His584 and Glu585 (hydrogen bonds are shown as dashed lines). (*c*) The +1 subsite of BT3130–ManI overlaid with MSM. The 580-loop enters the subsite from the left of the figure at a steeper trajectory; while His581 is conserved, no residue equivalent to the major coordinating side chain, Glu585, is present. A unique tryptophan pair at 172 and 198 offer potential for sugar binding. (*d*) +1 subsite overlay of BT3965–ManI with MSM. Equivalent 570-loop amino acids are conserved; however, these residues are more distantly located. Pro520 (clashing with MSM O6 in this figure), Trp526 and Tyr45 narrow the vertical dimension of the BT3965 binding cavity.

Structural alignment of BT3130 and BT3965 with a previously solved structure of BT3990 in complex with a non­hydrolysable substrate mimic, methyl α-d-manno­pyranosyl-2-thio-α-d-mannopyranoside (MSM; see Zhu *et al.*, 2010 ▸), reveals extensive diversity of the residues immediately adjacent to the catalytic centre (Fig. 4 ▸). In all three structures, when viewed from the exterior, looking along the scissile-bond axis, the −1 subsite appears directly posterior, with the +1 subsite immediately in front. Here, the boundary between these subsites can be delineated by a plane, running in the plane of the page, between the catalytic acid (Glu535, Glu531 and Glu533 in BT3130, BT3965 and BT3990, respectively), Cys399/392/393 and the interglycosidic S atom (see Fig. 4 ▸ *a*). Posterior to this plane, within the −1 subsite cavity, almost all major structural elements and protein–ligand interactions are highly conserved across diverse enzyme specificities. However, anterior to this plane, the residues and structural motifs that are responsible for coordinating the +1 carbohydrate moiety are highly variable. In BT3990, α-1,2-mannosidase activity appears to be conferred by a triad of amino-acid side chains interacting with the top face of the +1 moiety: hydrogen-bonding interactions between Glu585 and O3/O4 and between His584 and O3, and a hydrophobic interaction with Trp88, position the sugar ring within this region of the active site (Fig. 4 ▸ *b*). Interestingly, both the +1 anomeric substituent and O6 positions project outwards into solvent space, achieving minimal interaction with the protein (the nearest amino-acid side chain is >5 Å distant), suggesting that the enzyme is able to accommodate extended substrates featuring additional reducing-end sugars. Crucially, within this region neither the identified sugar-coordinating residues nor the major structural motifs upon which they are presented appear well conserved. Within BT3130, the 580-loop is positioned lower, and projects into the +1 subsite at a steeper angle, leaving a larger solvent-exposed space at the outer surface of the substrate-binding cavity. Furthermore, the hydrophobic ‘roof’ of this subsite is contributed by Trp67 (rather than Trp88 as in BT3990) and appears shifted some 2.5 Å towards the outer surface. In BT3130, the space occupied by Trp88 is replaced by a sterically modest Gly92–Ala93 motif positioned immediately adjacent to a stacked tryptophan pair formed by Trp172 and Trp198 (Fig. 4 ▸ *c*). Such aromatic platforms, arranged in a conformation that is likely to form π-stacking interactions above and below the plane of a sugar ring, may constitute an additional, distal binding subsite. Together, these observations are suggestive of a requirement for complex substrates that are capable of occupying an extended array of subsites within the BT3130 active site and possibly involving multiple additional sugars, consistent with the high activity of this enzyme against larger branched substrates such as yeast α-mannan, yet low activity towards simple disaccharides.

The +1 subsite of BT3965 is even more divergent than its counterparts. Structural features that could interact with the O3/O4 positions in the overlaid MSM complex are recessed, and no amino acids are identifiable that could contribute to the potential definition of a distal subsite, as has been proposed above for BT3130. Consequently, substantial lateral solvent space surrounds both sides of the +1 position, with few candidate side chains suggestive of a clear role in ligand binding (Fig. 4 ▸ *d*). Furthermore, BT3965 has variations within the structural elements that define both the roof and base of the active-site cavity. The bulky, hydrophobic side chains of Ile71, Pro520 and Trp526 project into the +1 subsite from above and below and are likely to be involved in positioning the reducing-end sugar moiety, while the side chain of Tyr45 appears well located to make a hydrogen-bonding interaction from above (Fig. 4 ▸ *d*). Collectively, these residues serve to narrow the vertical dimension within this region of the binding pocket relative to other family members.

Although shorter, and positioned approximately 2–3 Å distant, the 570-loop of BT3965 (equivalent to the 580-loop in BT3990) contains amino-acid side chains that appear capable of making analogous +1 subsite substrate-coordinating hydrogen bonds, most notably the side chains of His577 and Asp578. However, these residues are at substantially differing positions relative to the +1 moiety of the BT3990–MSM complex, suggesting that a different presentation of the +1 sugar would likely be required in order to bridge this gap and engage in hydrogen-bonding interactions. Similar to BT3130, unique structural features and rearrangements beyond the core catalytic pocket of BT3965 suggest that these residues may confer a tightly defined substrate specificity and thus biological role for this enzyme. BT3965 is one of only four characterized α-1,4-mannosidases in family GH92, a relatively unusual activity as α-1,4-mannoside linkages are comparatively rare in nature, featuring predominantly in fungal cell walls (Gorin *et al.*, 1977 ▸) and in the core structure of both human and fungal GPI protein anchors (Imhof *et al.*, 2004 ▸). Within these complex carbohydrate/glycolipid environments, individual sugar residues are often found with unusual modifications such as ethanolamine or phosphate groups, potentially leading to intricate substrate-binding requirements, and hinting at a possible role for these enzymes, while also lending an enticing explanation for the assortment of unique side chains present within the distal active-site cavity of BT3965.

The expansion and diversity of genes devoted singularly to carbohydrate processing/metabolism within gut-resident bacteria remains a remarkable and unique outcome of microbial evolution. Symbiotic glyco-specialists such as *Bt* maintain some of the largest and most sophisticated repertoires of CAZymes currently known, accounting for up to 10% of their protein-encoding genome (Xu *et al.*, 2003 ▸). This large concentration of superficially functionally similar genes appears to have arisen through the highly systematic fashion in which *Bt* and others organize and regulate their carbo­hydrate-processing machinery. Many gut microbes segregate and arrange such genes not according to biochemical/catalytic activity, but rather within functionally relevant PULs that encode the full set of enzymes necessary for the complete utilization of specific complex glycans (Martens *et al.*, 2009 ▸). These loci have evolved as discrete genetic modules, facilitating *en bloc* exchange among related bacteria, and are capable of sensing, binding, completely deconstructing and importing the component fragments of a given polysaccharide substrate, often all under the control of a single promotor element. Within *Bt*, several PULs have been shown to target polysaccharides rich in α-linked mannose sugars, including yeast cell-wall α-mannan and eukaryotic high-mannose-type N-glycans (Cuskin *et al.*, 2015 ▸). As might be expected, these PULs are populated by numerous genes belonging to α-mannoside-specific CAZy families, including GH38, GH76, GH92 and GH125, and result in apparent redundancy. While the *Bt* genome contains 23 GH92 genes, all encoding superficially similar exo-acting α-mannosidases, their distribution both within, and external to, several different PULs, as well as their diverse specificity for various substrates and/or linkages, strongly support individual and highly specialized biological functions for each. BT3965 is located within a PUL of unknown function that has been shown to be upregulated by both mannose-containing glycans (Sonnenburg *et al.*, 2006 ▸) and human milk oligosaccharides (Marcobal *et al.*, 2011 ▸), while BT3130 appears to be external to any currently characterized PULs. Our structural analysis shows that despite their distinct biochemical activities, both BT3130 and BT3965 share conserved core architectures, molecular mechanisms, TS^‡^ conformation and catalytic itineraries, strongly implying evolution from a common ancestral gene. Previous phylogenetic analysis (see Supplementary Fig. S7 in Zhu *et al.*, 2010 ▸) reveals that GH92 enzymes cluster into three broad clades. BT3130 and BT3965 are found together within the same clade, and are distinct from previously published structures of the α-1,2-specific enzymes BT3990 and BT2199. Interestingly, all enzymes within the BT3130/BT3965 clade are α-1,3-specific or possess multiple activities including α-1,3-mannosidase, with the exception of BT3965. This observation potentially suggests that evolution of α-1,4-mannosidase activity in BT3965 is a comparatively recent event, and that the broad structural similarity maintained with BT3130 is not coincidental. Nevertheless, that both enzymes maintain strong conservation of active-site structural features with the phylogenetically distinct BT3990 emphasizes the broad commonality of catalytic function and mechanism among *Bt* GH92 enzymes. The observation of a +1 subsite permitting high variability immediately adjacent to the catalytic centre reveals a genetic/biochemical mechanism through which *Bt* has been able to evolve a breadth of diverse enzyme specificities, tailoring activity to optimally metabolize various complex substrates, while still preserving a common route to catalysis. Intricacies in the fine specificities of members of a sequence-related family highlight the need for biochemical and structural studies to understand the functional roles of PULs, and how the composition of these gene cassettes can impact and inform on the overall health of the host. Knowledge of health-promoting gene/enzyme activities, together with genomic analyses of gut-microbial species, may in future reveal routes towards more effective, personalized treatments for chronic conditions such as diabetes, obesity and Crohn’s disease (reviewed in Kau *et al.*, 2011 ▸), all of which have been shown to have strong links to microbiota function.

## 4. Related literature   

The following references are cited in the Supporting Information for this article: Ashkenazy *et al.* (2016 ▸) and Landau *et al.* (2005 ▸).

## Supplementary Material

PDB reference: BT3130, 6f8z

PDB reference: complex with mannoimidazole, 6f90

PDB reference: BT3965, 6f91

PDB reference: complex with mannoimidazole, 6f92

Supplementary Figures.. DOI: 10.1107/S2059798318002942/jc5013sup1.pdf

## Figures and Tables

**Table 1:** Expression-construct/protein-production information

	BT3130	BT3965
Source organism	*B. thetaiotaomicron*	*B. thetaiotaomicron*
DNA source	Genomic DNA	Genomic DNA
Forward primer† ‡	CTCCAGCCATGGGC**CAGGCAGGGGAAATCACTAAATATG**	CTCCAGCCATGGGT**GCTCAAACTGAAAAGCTGAC**
Reverse primer‡ §	CTCCAGCTCGAG **CCATCCGGATGGTTTCTTACC**	CTCCAGCTCGAG **AAACCCAATTGATGATTTATAC**
Cloning vector	pET-21a	pET-21a
Expression vector	pET-21a-BT3130¶	pET-21a-BT3965¶
Expression host	*E. coli* Tuner (DE3)	*E. coli* Tuner (DE3)
Complete amino-acid sequence of the construct produced††	MGQAGEITKYVNPFIGTGAIDGGLSGNNYPGATSPFGMIQLSPDTSEAPNWGDASGYDYNRNTIFGFSHTRLSGTGASDLIDITLMPTSSGRTSSAFTHDEEKARPGYYQVMLKDENINAELTTTQRNGIHRYQYPAGKDAEIILDMDHSADKGSWGRRIINSQIRILNDHAVEGYRIITGWAKLRKIYFYMEFSSPILTSTLRDGGRVHENTAVINGTNLHGCFRFGQLNGKPLTCKVALSSVSMENARQNMEQEAPHWDFDRYVAAADADWEKQLGKIEVKGTEVQKEIFYTALYHTMIQPNTMSDVNGEYMAADYTTRKVANNETHYTTFSLWDTFRASHPLYTLLEPERVTDFVKSMIRQYEYYGYLPIWQLWGQDNYCMIGNHSIPVITDAILKGIPGIDMEKAYEAVYNSSVTSHPNSPFEVWEKYGFMPENIQTQSVSITLEQAFDDWCVAQLAAKLNKDADYQRFHKRSEYYRNLFHPKTKFFQSKNDKGEWIEPFDPYQYGGNGGHPFTEGNAWQYFWYVPHNIQALMELTGGTKAFEQKLDTFFTSTYKSEQMNHNASGFVGQYAHGNEPSHHVAYLYNFAGQPWKTQKYVSHILNTLYNNTSSGYAGNDDCGQMSAWYVFSAMGFYPVNPADGRYIIGSPLLDECTLKLAGNKEFRIRTIRKSPEDIYIQSVTLNGKKHKDFFITHQDIMNGGTMVFKMGKKPSGWLEHHHHHH	MGAQTEKLTDYVNPFVGTDGYGNVYPGAQIPFGGIQISPDTDSRFYDAASGYKYNHLTLMGFSLTHLSGTGIPDLGDFLFIPGTGEMKLEPGTHEDPDQGYRSRYSHDKEWASPNYYAVELADYGVKAEMTSGVRSGMFRFTYPESDNAFIMIDMNHTLWQSCEWSNLRMINDSTITGYKLVKGWGPERHVYFTATFSKKLTGLRFVQDKKPVIYNTSRFRSSYEAWGKNLMACISFDTKAGEEVTVKTAISAVSTDGARNNMKELDGLTFNELRAKGEALWEKELGKYTLTADRKTKETFYTSAYHAALHPFIFQDSDGQFRGLDKNIEKAEGFTNYTVFSLWDTYRALHPWFNLVQQEVNADIANSMLAHYDKSVEKMLPIWSFYGNETWCMIGYHAVSVLADMIVKEVKGFDYERAYEAMKTTAMNSNYDCLPEYREMGYVPFDKEAESVSKTLEYAYDDYCIAQAAKKLGKEDDYHYFLNRALSYQTLIDPETKYMRGRDSKGDWRTPFTPVAYQGPGSVHGWGDITEGFTMQYTWYVPQDVQGYINEAGKELFRKRLDELFTVELPDDIPGAHDIQGRIGAYWHGNEPCHHVAYLYNYLKEPWKCQKWIRTIVDRFYGNTPDALSGNDDCGQMSAWYMFNCIGFYPVAPSSNIYNIGSPCAEAITVRMSNGKNIEMTADNWSPKNLYVKELYVNGKKYDKSYLTYDDIRDGVKLRFVMSGKPNYKRAVSDEAVPPSISLPEKTMKYKSSIGFLEHHHHHH

† Underlined regions indicate NcoI restriction sites.

‡ Primer sequences in bold represent overlap/homology regions to the gene of interest.

§ Underlined regions indicate XhoI restriction sites.

¶ Genes were cloned from genomic DNA immediately into appropriate vectors for expression.

†† Underlined amino acids indicate vector-added residues, including a C-terminal His_6_ tag.

**Table 2:** BT3130 and BT3965 crystallization conditions

	BT3130	BT3965
Method	Hanging-drop vapour diffusion	Hanging-drop vapour diffusion
Plate type	24-well tissue-culture plate	24-well tissue-culture plate
Temperature (K)	292	292
Protein concentration (mg ml^−1^)	23.8	56.0
Buffer composition of protein solution	50 m*M* HEPES pH 7.0, 300 m*M* NaCl, 10 m*M* calcium acetate	50 m*M* HEPES pH 7.0, 300 m*M* NaCl
Composition of reservoir solution	18%(*w*/*v*) PEG 3350, 0.1 *M* bis-tris propane pH 6.4, 0.2 *M* NaBr	20%(*w*/*v*) PEG 3350, 0.2 *M* NaNO_3_
Volume and ratio of drop	3 µl (2:1)	3 µl (2:1)
Volume of reservoir (µl)	500	500

**Table 3:** Data collection and processing for BT3130 Values in parentheses are for the outer shell.

	Native BT3130	BT3130–ManI
Diffraction source	Beamline I04-1, DLS	Beamline I03, DLS
Wavelength (Å)	0.92000	0.97950
Temperature (K)	100	100
Detector	PILATUS 2M	PILATUS 6M
Crystal-to-detector distance (mm)	249.53	388.49
Rotation range per image (°)	0.2	0.2
Total rotation range (°)	220	220
Exposure time per image (s)	0.1	0.1
Space group	*P*6_2_22	*P*6_2_22
*a*, *b*, *c* (Å)	272.3, 272.3, 190.0	273.7, 273.7, 189.8
α, β, γ (°)	90.0, 90.0, 120.0	90.0, 90.0, 120.0
Mosaicity (°)	0.08	0.08
Resolution range (Å)	49.67–2.50 (2.54–2.50)	49.38–2.40 (2.44–2.40)
Total No. of reflections	3551261	4028427
No. of unique reflections	142171	160656
Completeness (%)	99.8 (96.7)	99.5 (99.2)
Multiplicity	25.0 (24.5)	25.1 (25.9)
〈*I*/σ(*I*)〉	18.6 (2.2)	18.4 (1.9)
*R*_r.i.m._	0.218 (2.093)	0.203 (2.586)
Overall *B* factor from Wilson plot (Å^2^)	33.4	40.1

**Table 4:** Data collection and processing for BT3965 Values in parentheses are for the outer shell.

	Native BT3965	BT3965–ManI
Diffraction source	Beamline I04, DLS	Beamline I04-1, DLS
Wavelength (Å)	0.97949	0.92000
Temperature (K)	100	100
Detector	PILATUS 6M	PILATUS 2M
Crystal-to-detector distance (mm)	339.63	221.06
Rotation range per image (°)	0.2	0.2
Total rotation range (°)	220	220
Exposure time per image (s)	0.1	0.1
Space group	*P*2_1_	*P*2_1_
*a*, *b*, *c* (Å)	111.9, 184.5, 183.7	82.5, 186.9, 95.1
α, β, γ, (°)	90.0, 90.8, 90.0	90.0, 91.7, 90.0
Mosaicity (°)	0.13	0.12
Resolution range (Å)	48.08–1.80 (1.83–1.80)	46.73–1.90 (1.93–1.90)
Total No. of reflections	2840362	945318
No. of unique reflections	682384	216402
Completeness (%)	99.5 (95.8)	96.1 (96.4)
Multiplicity	4.2 (4.1)	4.4 (4.3)
〈*I*/σ(*I*)〉	10.4 (1.4†)	7.1 (1.4†)
*R*_r.i.m._	0.113 (1.246)	0.200 (1.225)
Overall *B* factor from Wilson plot (Å^2^)	16.9	14.7

† Data were processed to CC_1/2_ > 0.5, outer shell completeness of >95%.

**Table 5:** Structure solution and refinement for BT3130 Values in parentheses are for the outer shell.

	Native BT3130	BT3130–ManI
Resolution range (Å)	49.30–2.50	49.40–2.40
Completeness (%)	100.0	99.4
σ Cutoff	†	†
No. of reflections, working set	135416	153712
No. of reflections, test set	7030	8000
Final *R*_cryst_	0.181	0.207
Final *R*_free_	0.222	0.242
No. of non-H atoms		
Protein	16683	16434
Ion	3	3
Ligand	0	42
Solvent	1297	1186
Total	17983	17665
R.m.s. deviations		
Bonds (Å)	0.0092	0.0087
Angles (°)	1.318	1.267
Average *B* factors (Å^2^)		
Protein	52.4	65.5
Ion	63.3	63.5
Ligand	0	65.9
Solvent	49.2	55.5
Ramachandran plot		
Most favoured (%)	96.2	96.1
Allowed (%)	3.3	3.6

† No σ cutoff was applied during refinement.

**Table 6:** Structure solution and refinement for BT3965 Values in parentheses are for the outer shell.

	Native BT3965	BT3965–ManI
Resolution range (Å)	48.10–1.80	46.70–1.90
Completeness (%)	99.5	96.0
σ Cutoff	†	†
No. of reflections, working set	651952	214199
No. of reflections, test set	34061	11179
Final *R*_cryst_	0.160	0.176
Final *R*_free_	0.185	0.208
No. of non-H atoms		
Protein	48139	24408
Ion	24	20
Ligand	0	56
Solvent	6876	2321
Total	55039	
R.m.s. deviations		
Bonds (Å)	0.0123	0.0105
Angles (°)	1.527	1.421
Average *B* factors (Å^2^)		
Protein	27.1	23.3
Ion	28.6	23.8
Ligand	0	20.0
Solvent	39.2	28.8
Ramachandran plot		
Most favoured (%)	97.2	97.0
Allowed (%)	2.6	2.9

† No σ cutoff was applied during refinement.
