# Supplementary material for: Bacteroides thetaiotaomicron generates diverse α-mannosidase activities through subtle evolution of a distal substrate-binding motif
Source: Acta Crystallogr D Struct Biol. 2018 Apr 24;74(Pt 5):394–404. doi: 10.1107/S2059798318002942 (PMC5930347; doi:10.1107/S2059798318002942)
Supplement: Supplementary file 1 [file d-74-00394-sup1.pdf]

STRUCTURAL
BIOLOGY

Volume 74 (2018)

Supporting information for article:

Bacteroides thetaiotaomicron generates diverse α-mannosidase activities through subtle evolution of a distal substrate-binding motif

Andrew J. Thompson, Richard J. Spears, Yanping Zhu, Michael D. L. Suits, Spencer J. Williams, Harry J. Gilbert and Gideon J. Davies

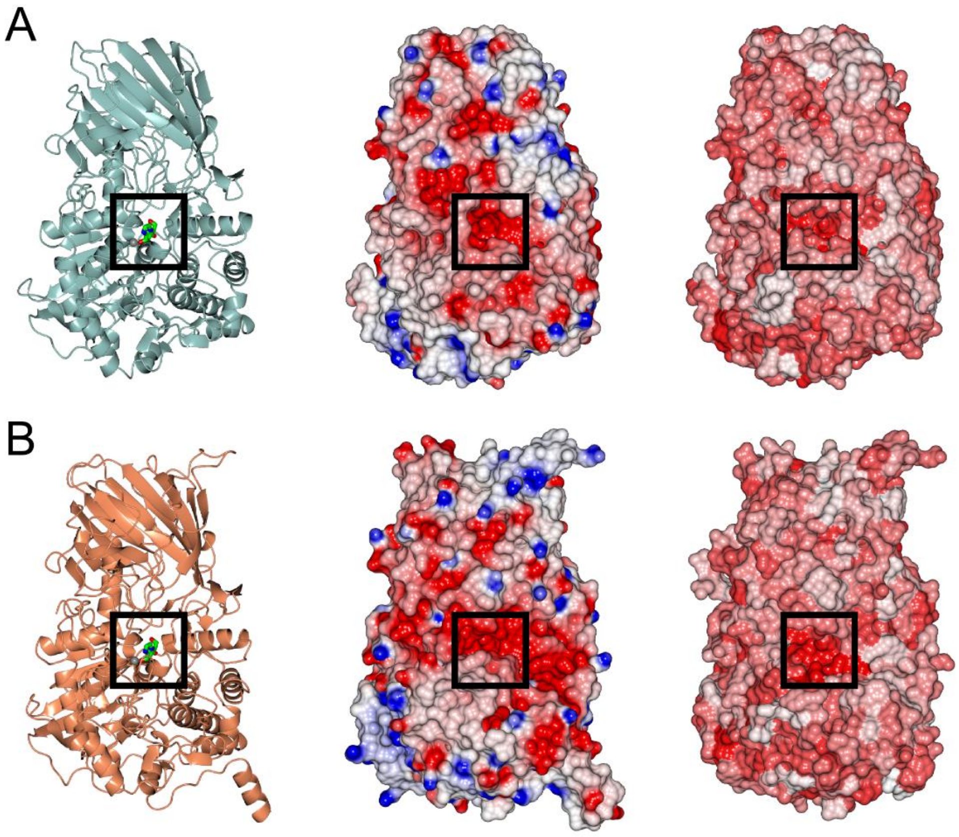

Figure S1 Alternate structural representations of BT3130 (A, green ribbons) and BT3965 (B, orange ribbons). Panels, from left to right, show ribbon projections of respective enzyme-ManI complexes (ManI and a bound Ca^{2+} ion are drawn as enlarged cylinders and spheres respectively), enzyme-ManI contoured as an electrostatic surface, and finally native enzyme structures coloured according to sequence conservation using the expanded alignment shown below in Figure S3 (areas of strong conservation are indicated by intense red shading). The location of the active site pocket is indicated by a dark box in each model. While amino acids in both molecules are generally well conserved, strongest similarity is observed within the inner core of the substrate binding pocket. Sequence alignments were mapped to coordinate files using the Consurf server (Ashkenazy *et al.*, 2016, Landau *et al.*, 2005).

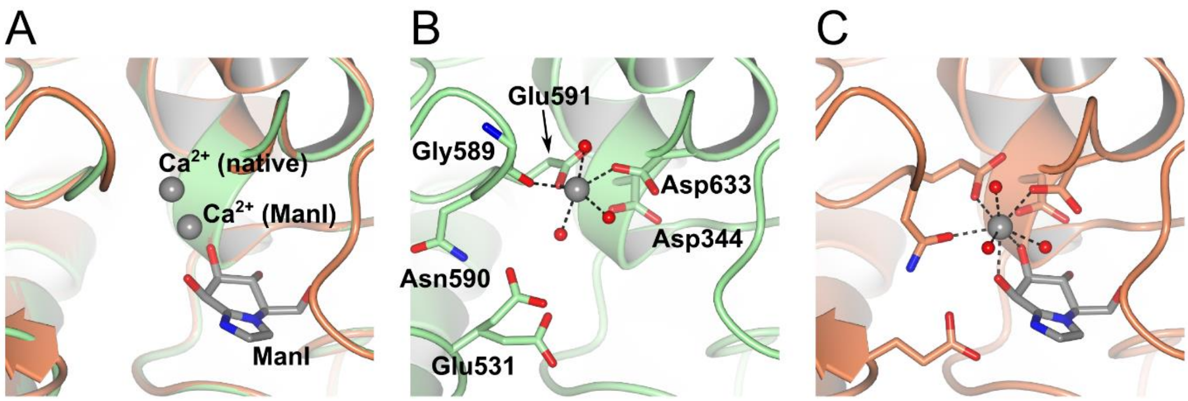

Figure S2 Overlay (A) of Ca^{2+} bound in the active site of BT3965-native (light green, and panel B) and BT3965-ManI (orange, and panel C). Ca^{2+} is aberrantly coordinated in native BT3965 (B), and although both clear octahedral coordination geometry and an observed strong difference map peak in the absence of a modelled ion are suggestive of Ca^{2+} binding, *B*-factor analysis compared to surrounding atoms suggests only approximately 50-60% occupancy at this site. Displacement of Ca^{2+} away from the ideal position for coordination and catalysis yields a dual, unnatural conformer in the catalytic acid, Glu531. Supplementation with exogenous divalent salts and binding to ManI (C) brings about a subtle conformational change, rotating Asn590 into the active site pocket, positioning both Ca^{2+} and Glu531 into the activated state.

```
BT1032      ---MRR------ILLTCTLAFTLLPVFGGEGKAPPMDKEKSLLIDYVDPFIGTT------
BT1769      -------MKLTHILASAAIISTLSA--CNG----SLQTADRTPVDYVNPYIGNISH----
BT1878      MIIMKRKSPVMAV---ALAGLFMYTS-CSP----TEQAETKDYTQYVNTFIGAA------
BT2199      ---MVTR---LFFLSIC--FPFLLTS-CQ------QSKKTVEFVDYVNPLMGTESTFA--
BT2629      ---MKTHFSFKHLLFLG--GAVL--Y-SL------QSSAVKNPVDYVSTLIGTQSKFE--
BT3130      ----------MKK---LLLSVCAFSL-TLA------TLQAGEITKYVNPFIGTGAIDG--
BT3527      ---------MKKLALLA--FTLFSAW-NM------DAKTITGPVDYVSPLVGTQSKHA--
BT3773      -------MNAFKL---LMAASCFLGL-ATSGVAKQDYSKSEGLLQYVDPYIGSG------
BT3784      ---MKTHFSFKHLLFIG--GAVL--Y-SM------QISAVKNPVDYVSTLVGTQSKFE--
BT3858      --MMMNRLNIKRTVGSCLMAMAFFS--CTH----TDQTPTKDFVDYVNPYIGNISH----
BT3962      -------MNKQKLLSIT--MTLLLG--VS------SIFAQKQPVDYVNPLMGTDSKIS--
BT3963      -------MNKMKK------KVLMMTM-AAV----TLSAVAQQPVDYVNPIIGTN------
BT3965      ------------------------------------MAQTEKLTDYVNPFVGTD------
BT3990      ---------MKRLLLIA--CILNCTL-LS---------QAKDWTQYVNPLMGSQSTFE--
BT3991      -------MKLKTLLIGCFGGFIMLNS-CTE------SPSVKDYSAYVNPFIGTG------
BT3994      -------MKTPIY---LLLIVCIFAS-CNT----KQTQAEIDYTSYVNPFIGTD------
BT4073      -------MYKASANILIACSFLWLTA-CSSA---GVVTEDLIPTDYVNPFIGASTSVGAA
BT4092      ---------MKKLVYLI--IFFLY-S-TV------MNAQLKDLVQYVNTLQGTDSNFG--
BT4093      ---MK----HK-FLFILLFSLVLEGM-V------TTQAVAGDYVHQVNTLIGTKGTG---
                                                          *.   *

BT1032      ----NFGTTNPGAVCPNGMMSVVPFNVMG--SADNTYDKDARWWSTPYEHTNCFFTGYSH
BT1769      ----LLVPTFPTIQLPNSMLRVYPERAD---------------------YTSELLKGLPL
BT1878      ----DNGHTFPGACYPFGMIQTSPVT-GAV----------GWRYCSEYTYQDSLIWGFTQ
BT2199      ---FSHGNTYPAVAVPWGMNFWSPQTGEN-----------GSGW--MYTYTDSLMRGFRQ
BT2629      ---LSTGNTYPATALPWGMNFWTPQTGKM-----------GDGW--AYTYDADKIRGFKQ
BT3130      ---GLSGNNYPGATSPFGMIQLSPDTSEAP----------NWGDASGYDYNRNTIFGFSH
BT3527      ---LSTGNTYPAIALPWGMNFWVPQTGKM-----------GDGW--AYTYDADKIRGFKQ
BT3773      ----FHGHVFVGTSVPYGMVQLGPNN-IHK----------GWDWCSGYHYSDSILIGFSH
BT3784      ---LSTGNTYPATALPWGMNFWTPQTGKM-----------GDGW--AYTYNADKIRGFKQ
BT3858      ----LLVPTYPTVHLPNSMLRVYPERGD---------------------YTSDRVNGLPV
BT3962      ---LSNGNTYPAIALPWGMNFWMPQTGKM-----------GDGW--AYTYASDKIRGFKQ
BT3963      ----GMGHTFPGACTPFGWVQLSPDTDTIPHNVNGAYQKNAYEYCAGYQYRDKTIVGFSH
BT3965      ----GYGNVYPGAQIPFGGIQISPDTDSR-----------FYDAASGYKYNHLTLMGFSL
BT3990      ---LSTGNTYPAIARPWGMNFWTPQTGKM-----------GDGW--QYTYTANKIRGFKQ
BT3991      ----GHGHTFPGAVVPHGMIQPSPDT-RID----------GWDACSGYYYDDNTINGFSH
BT3994      ----FTGNTYPGAQAPFGMVQLSPDN-GLP----------GWDRISGYFYPDSTIAGFSH
BT4073      GVYHGLGKTFPGATTPYGMVQVSPNTIT------------GGDNSSGYSDEHKTIEGFAF
BT4092      ---LSYGNTYPTTGMPYGMHMWSAQTGKN-----------GDGF--KYMYAVDKIRAFSQ
BT4093      ---LTSGYLYPGATYPYGMVQFTPSYFS-------------------------KRSGFVI
                           * .                                      .

BT1032      VNLSGVGC-PELGSLLLMPTTGELNVD-------YKEYGSKYK--DEQASPGYYSNYLTK
BT1769      IVTNHRE----RSAFNFSPYQGEKLR-----------PVITYNYDNEHITPYSFDVELDD
BT1878      THLNGTGC-MDLGDILVMPVTGTRAR-A----WD--AYRSHFPKDKEAATPGYYTVELSD
BT2199      THQPSPWI-NDYGTFSIMPLAGELKMS-------HKERLVPFSHQQEKATPYNYSVTFN-
BT2629      THQPSPWM-NDYGQFAIMPITGGLVFD-------QDRRASWFSHKAEVAKPYYYKVYLAD
BT3130      TRLSGTGA-SDLIDITLMPTSSGR-------------TSSAFTHDEEKARPGYYQVMLKD
BT3527      THQPSPWI-NDYGQFSIMPITGKAVFD-------QDQRASWFSHKAETATPYYYKVYLAD
BT3773      THLSGTGC-TDLGDILIMPLNEIRTP-RGNQDDIRDGYASKYSHDNEIARPEYYSLILDR
BT3784      THQPSPWM-NDYGQFSIMPITGGLVFD-------QDQRASWFSHKAEVAKPYYYKVYLAD
BT3858      VVTSHRG----SSAFNLSPVQGEVSR-----------PIVSYSYDLENITPYSYSVYLDE
BT3962      THQPSPWI-NDYGQFSIMPMTKQLKID-------QDSRASWFSHKAEKATPYYYSVYLSE
BT3963      THLSGTGH-SDLGDILLMPAVGDVKLNPGRADHPEEGYRSRFDHATEKATPGYYEVMLDD
BT3965      THLSGTGI-PDLGDFLFIPGTGEMKLEPGTHEDPDQGYRSRYSHDKEWASPNYYAVELAD
BT3990      THQPSPWI-NDYGQFSIMPIVGQPVFD-------EEKRASWFAHKGEVATPYYYKVYLAE
BT3991      THVSGTGC-CDYGDVLLMPTVGKPQYLTTDPESQKLAYASAFSHENETAEPGYYSVFLDT
BT3994      THLSGTGA-GDLYDISFMPVTLPYKE-A----EAPLGIYSKFSHDEESAYAGYYQVRLKD
BT4073      TQMSGVGWFGDLGNFLVMPTTGELQKIAGKEDGSIKGYRSSYDKATETAKAGYYSVELTD
BT4092      SHQCSPWV-SDYAVYSFMPMVGELVVN-------QDARATKFSHDNEIAKPHYYKVTFD-
BT4093      NQLSGGGC-EHMGNFPTFPVKGKLKMSPDN----I--LNYRINVSEEKGHAGYYEAMVQ-
                              *                           *      :   .

BT1032      YNIKTEVSATPRTGIARFTFPKGES-HILLNLGEGLTNES-----------GAMLRRV-S
BT1769      NRMKAEYALSHQSAIYRITYEADKPAYLIVNSRNG--------------------SIHAN
BT1878      PQVKAELTASIHAALHRYTYHKADSASLLIDLQHG---PAWREEQYHSQVNSCEVNWE-D
BT2199      NGLQTSLSATSRGAVFEVSFPEKEDQYVVVDAYNG---G-------------SSITIEPE
BT2629      HDVTTELAPTERAVMFRFTYPETKNAYVIVDAFDK---G-------------SYVKVIPE
BT3130      ENINAELTTTQRNGIHRYQYPAGKDAEIILDMDHSADKGSWGRRIINSQI-----RIL-N
BT3527      HDVVTEIAPTERAAAFRFTFPENDHSYVVVDAFDN---G-------------SFVKVIPS
BT3773      YNIKAELTATDRVGFHRYTYPEGKPVSILIDLREGNGSNAY-----DSYI-----RKV-D
BT3784      HDVTTELVPTERAAMFRFTYPETKNAYVVIDAFDK---G-------------SYVKVIPE
BT3858      ADIQVEYAPSHQAGIYHISFGTEGDNALVVNTKNG--------------------KLVAE
BT3962      YNMTTEIAPTERCAYFRFTFPEASDAYVVVDAFDR---G-------------SYVKVIPE
BT3963      YGIKAQLTATQRTGVHKYTFPKGKDGHLILDLVHGIY-------NYDGKVLWANLRVE-N
BT3965      YGVKAEMTSGVRSGMFRFTYPESDNAFIMIDMNHTLW-------Q---SCEWSNLRMI-N
BT3990      HDIVTEMTPTERAVLFRFTFPENDHSYVVVDAFDK---G-------------SYIKIIPE
BT3991      YQVKAEITATKRGAIHRYTFPESTESGFIIDLDYSLQRQ----TNYE-----MEIEVI-S
BT3994      YHINVELTATERCGIQRYTFPKAEAAI-FLNLKKA---MNWD-FTNDSHI-----EVV-D
BT4073      YKIKVESSATPHCGILQFTFPSNEQSRIQIDLARRVGGTS----------TSQYVKVL-D
BT4092      NGITTEMAPTTRGVHLRFSYPTTGDAYLVLDGYTD---M-------------SEIKIDPA
BT4093      EDIKAKLTVTERTGMASYEYPADQQYGTIIIG-GGISAT---------PIEQAA-IVITA
              : ..     :       :         :

BT1032      DSEIEGMKLL----GTFCY-NPQAVFPIYFVMRVNKVPATTGYWKKQRPMTGVEAEWDRD
BT1769      ENFISGRQQL----N--------DNTNVYVYIEAQEKPISAGILENGTIET---------
BT1878      AQTLAGHVNN----TVWV------DQDYFFVMKFNRPVIDSLYLPMGET-----------
BT2199      KRLVKGATRY----NN---GGVPDNFANYFMMEFSHPVIEYGTYNGD--TL---------
BT2629      ENKIIGYSTK----NS---GGVPENFKNYFVIQFDKPFTFVSTVFEN--NI---------
BT3130      DHAVEGYRII----TGWAK-----LRKIYFYMEFSSPILTSTLRDGGRV-----------
BT3527      ENKIIGYTTK----NS---GGVPANFKNYFVLEFDKPFTYTAAVANG--NI---------
BT3773      DYTVEGYRYV----RGWSP-----SRKVYFVLKSDQKIEKFTAYDDNTPKL---------
BT3784      ENKIIGYSTK----NS---GGVPENFKNYFVIQFDKPFTFTSGVKEN--NI---------
BT3858      EKGVSGYQVI----DN-------TPTKIYLYLETSQLPLRKGVLADGKVDM---------
BT3962      ENKIVGYTTR----NS---GGVPQNFRNYFVIEFDKPFTFNKVWADY--HL---------
BT3963      DTLLTGYRIT----NGWAR-----TNYTYFAISLSQPIKDYGYKDK-EKVLYNGF-W---
BT3965      DSTITGYKLV----KGWGP-----ERHVYFTATFSKKLTGLRFVQDKKPVIYNTS-R---
BT3990      ENKIIGYTTR----NS---GGVPENFKNYFIIEFDKPFTYKATVENG--NL---------
BT3991      DTEICGHKKT----TYWAF-----DQYINFYAKFSKPFAYTLITDSVTM-----------
BT3994      SVTIQGYRYS----DGWAR-----DQRIYFRTRFSKPFDKVELDTTAII-----------
BT4073      DYTIQGWMKCTPDGGGWGNGEGNSDYTVYYYAQFSKPLSNYGFWSADIPDEWVRK-RDEV
BT4092      KRQISGWVNN----QR--FVNDSKSFRNYFVVQFDKPFEDYGVWENQKDEV---------
BT4093      PNKCEGYAEG----GNF--CGLRTPYKVYFVAEFDTDAFETGTWKREEL-----------
                 *                            .

BT1032      --------QGKYKLYTRYGKEIAGDD---VGAYFTFETEEGEQVEVQMGVSFVSIENARL
BT1769      -----------------SKDNAEGAN---ACAAWRFA-DGTTTVNLRYGISFISEEQAEK
BT1878      ---------------------EKGKR---IIATF--DLKPGDELMMKVALSTTSVEGAKK
BT2199      ---------------LHHQTDVAADY---TCAYLKFDVPAGEKLTIRTASSFISPEQAAI
BT2629      ---------------LPNETEAKGNH---TGAVIGFATKKGEIVHARVASSFISPEQAEL
BT3130      ---------------HENTAVINGTN---LHGCFRFGQLNGKPLTCKVALSSVSMENARQ
BT3527      ---------------DTNKLEANDKH---AGALIGFKTRKGEQVNVRVASSFISPEQAEL
BT3773      ---------------WDQLKV---ES---VKSVLTF--GNVKEVKIKVAISSVSCDNAAM
BT3784      ---------------LPNETEVQGNH---TGAIIGFATQKGEIVHARVASSFISYEQAEL
BT3858      -----------------ESKEG--------SAIALYY-GSEKNLNLRYGISFISAEQAKK
BT3962      ---------------VETHLELQSNH---VGAAIGFSTKKGEQVHAKVASSFISPEQAEL
BT3963      ---------RRFKL-EKNFPEITGRK---IVAYFNFETAKDPELVVKVALSAVSTEGAVK
BT3965      ---------FR------SSYEAWGKN---LMACISFDTKAGEEVTVKTAISAVSTDGARN
BT3990      ---------------QENVAEQTTDH---AGAIIGFKTRKGEQVNARIASSFISFEQAAA
BT3991      ---------------------DNGKRLPVCKAVLHFNTKKDEEVLVKVGVSAVDIAGARK
BT3994      ---------------KDKQ--HIGTA---VIARFDFHTEEGEQILVNTAISGVSMEGAAK
BT4073      VSIPYLTRISQAPV-IKDKKELEGKH---LGFFTEFPTKEGEQVEMKVGISFVDMEGAAN
BT4092      ---------------FPQKLDGAGKG---YGAFIKF--KKGSKVQAKAASSYISAEQALI
BT4093      ---------------MPNTTFAEGEY---SGVYFTFDVNKKKNIQYKIGVSYVSVENARE
                                                       :  . . *  .   *
```

```
BT1032          NLDKEQSGK-NFEQVLSDARAQWNDDLSRITVEGGTDAQKTVFYTALYHLLIHPNVLQDV
BT1769          NLHRELKDY-NIKALAEAGRQIWNETLGRIQVEGGTEDDKTVFYSSFYRTFERPICMSEG
BT1878          NLQAEIPDW-NFDGVKLAAHDEWNSYLSRMDVEGTDDE-KTNFYTCFYHALIQPNQISDV
BT2199          NFNREVADA-DVQLISGKAREQWNNYLGRVEAEGGTDEQLRTFYSCLYRTLLFPREFYEF
BT2629          NLKE-LGKN-SFDQLVANGREIWNREMSKIEIEDDNIDNLRTFYSCLYRSMLFPRSFYEI
BT3130          NMEQEAPHW-DFDRYVAAADADWEKQLGKIEVKGTEVQ-KEIFYTALYHTMIQPNTMSDV
BT3527          NLKE-LGKD-NIDQIAAKGRKVWNDVLGRIEVEDDDIDHLRTFYSCLYRSVLFPRSFYEI
BT3773          NLQAELSHW-DFDKVVKMSSDRWNKQLDKMTVESDDEAAKRVFYTAHYHTMIAPTLYCDV
BT3784          NLKE-LGKD-SFDQLVTKGKDIWNREMSKVDVEDDNIDNLRTFYSCLYRSMLFPRSFYEI
BT3858          NLQRDITTY-DVKAVADAGRRIWNKTLGKIVIEGGSEDEKEIFYTSLYRTYERMINLSED
BT3962          NLKE-IGNK-TFEQTKEAGRKAWNDVLGRIKVEDDDENRMRTFYSCLYRSVLFPRMFHEV
BT3963          NLRAEASGK-SFEQLAEAARTDWNNELDHFEIEGTPDQ-KAMFYTSLYHTMINPSVYMDV
BT3965          NMK-ELDGL-TFNELRAKGEALWEKELGKYTLTADRKT-KETFYTSAYHAALHPFIFQDS
BT3990          NMNE-LGKD-NIEQLAQKGKDAWNQVLGKIEVEGGNLDQYRTFYSCLYRSLLFPRKFYEL
BT3991          NVESEIPEW-DFDKVRKDARTAWNNYLSKIDITTSDKEDKTIFYTALYHTAISPNLFTDA
BT3994          NLQAEVPEN-DFDKYLAETKANWNRQLGKIEVEGDNQDDKVNFYTALYHSMIAPTIYSDV
BT4073          NFKQEIASK-NFAQVKQEASDLWNKELSRIRISGGTDDEKTVFYTSLYHTMIDPRIYTDV
BT4092          TLNKELGKDKNLEVTKARGQKTWNEVLNRIVVEGCTDEQMKTFYSCLFRANLFSRKFYER
BT4093          NLKAENAEW-DFQKIQNQAEAKWNHYLGMIEVEGTNPDRTTQFYTHLYRSFIHPNVCSDV
                ..         .          *:  :.              **:  ::         :

BT1032          NGEYPA-----MESDQIL-TTKGTRYTVFSLWDTYRNVHQLLTLVYPERQMEMVRTMLDM
BT1769          -----GRYFSAFDGKVHE-DNGTPFYTDDWIWDTYRAAHPLRTLIDQQKEEDIIASYLRM
BT1878          DGMYRN-----AADSIVK-AGTGTFYSTFSLWDTYRAAHPFYTLMVPERVDDFVNSLIEQ
BT2199          DSQGNPVYYSPYDGNVH----DGYMYTDNGFWDTFRAVHPLFTLLYPEVSERVTQSIINA
BT2629          DAKGQVMHYSPYNGEVR----PGYMFTDTGFWDTFRCLFPFLNLMYPSMNQKMQEGLVNT
BT3130          NGEYMA-----ADYTTRKVANNETHYTTFSLWDTFRASHPLYTLLEPERVTDFVKSMIRQ
BT3527          DAKGDIVHYSPYNGEVL----PGYMFTDTGFWDTFRCLFPFLNLMYPSMNMKMQEGLVNT
BT3773          NGEYRG-----MNDMIYT-DPKKANYTTLSLWDTYRALNPLMTIIQPEMVDNVINSMLSI
BT3784          DAKGQVVHYSPYNGKVL----PGYMFTDTGFWDTFRCLFPFLNLMYPSMNQKMQEGLVNA
BT3858          -----GKYYSAFDGKIHE-DGGVPFYTDDWIWDTYRATHPLRILIEPQKELDMIRSYIRM
BT3962          NAKGETVHYSPYNGEIR----PGYMFTDTGFWDTFRCLFPFVNLIYPSMGEKMQEGLLNT
BT3963          DGSYRG-----LDHNIHQ-AKGFTNYTIFSLWDTYRAEHPFLNLVKPERNADMVESMIKH
BT3965          DGQFRG-----LDKNIEK-AEGFTNYTVFSLWDTYRALHPWFNLVQQEVNADIANSMLAH
BT3990          DANGQPIHYSPYNGQVL----PGYMFTDTGFWDTFRCLFPLLNLMYPSVNKEMQEGLINT
BT3991          DGRYLG-----MDLEVHQGDTINPLYTVFSLWDTFRALHPLMTIIDPNLNNQFINSLIRK
BT3994          DGAYYG-----PDKKVHQ-SDGWVNYSTFSLWDTYRAAHPLFTYTEPERVNDMVKSFIAF
BT4073          DGRYIG-----GDKKVHEQDGTFTKRTIFSGWDVFRSQFPLQAMINPRLVSDALNSLITM
BT4092          KENGEPYYYSPYNGKIY----DGYMYTDNGFWDTFRSQFPLTNILHPTMQGRYMNALLAA
BT4093          NGEYMG-----ADFRVHK--SRSKHYTSFSNWDTYRTQIQLLSMLDPEVASDIVISHQLF
                                          :    **.:*

BT1032          YRE--HGWFPKWELYGRETLTMEGDPSIPVIVDTWMKGLRDFDVDLAYEAMYKSATLP--
BT1769          AEQMGNMWMPTFPEVTGDTRRMNSNHAVATVADALAKGLK-VDTAKAYEACRKGIEEKTL
BT1878          GEV--QGFLPIWALWGKENFCMIGNHGVSVIAEAYRKGFRGFDAERAFNMVKKTQTVS--
BT2199          YNE--SGFMPEWASPG-HRGCMIGNNSVSLLVDAWMKGIQTVDAEKALEAMIHQTQAR--
BT2629          YKE--SGFLPEWASPG-HRDCMVGNNSASVVADAYIKGLRGYDIETLWEALKHGANA---
BT3130          YEY--YGYLPIWQLWGQDNYCMIGNHSIPVITDAILKGIPGIDMEKAYEAVYNSSVTS--
BT3527          YKE--SGFLPEWASPG-HRGCMVGNNSASVVADAYLKGLKGYDIETLWEAVKHGANAV--
BT3773          YRQ--QDKLPIWPLMSGETNCMPGYSSVPVIADAYLKGFTGFDAEEALTAMKATATYE--
BT3784          YLE--SGFLPEWASPG-HRDCMVGNNSASVVADAYIKGLRGYDIETLWEALKHDANA---
BT3858          AEQSDRRWMPTFPEVTGDSHRMNGNHAVAVIWDAYCKGLKDFDLEAAYEACKGAITEKTL
BT3962          YLE--SGFFPEWASPG-HRGCMVGNNSASVVADAFMKNVTKADAEKMYEGLLKGANSV--
BT3963          EQQSVHGMLPIWSLMGNENWCMSGYHAVSVLADAITKGVFSN-VDEALSAMVSTSTVP--
BT3965          YDKSVEKMLPIWSFYGNETWCMIGYHAVSVLADMIVKEVKGFDYERAYEAMKTTAMNS--
BT3990          YLE--SGFFPEWASPG-HRGCMVGNNSASILVDAYMKGVKVDDIKTLYEGLIHGTENV--
BT3991          HQE--GGIYPMWDLASNYTGTMIGYHAVPVIVDAYMKGYRNFDAHEAYKASLRAAEYD--
BT3994          FEQ--NGRLPVWNFYGSETDMMIGYHAVPVIVDAYLKGIGNFDAEKALAACVATANL---
BT4073          ADQSRREYYERWELLNSYSGCMIGNPALSVLADAYMKGIRTYDVEKAYQYAVNTSAKF--
BT4092          QEQ--CGWLPSWSSPG-ETGGMLGNHSISLLADAWAKGIRDFDPEKALKAYAHEAMNK--
BT4093          AEQ-SGGSFPRWVMANIETGVMQGDPTPILIANAYAFGARNYDPKPIFKIMRKGAEEP--
                           :         * .      : :

BT1032          GAE---------NLMRPDNDDYMSKGYVPLREQ--------Y-----DNSVSHALEYYIA
BT1769          APWSGAPAGWL-------DNFYRENGYIPALRVDEPENDPNVHPFEKRQPVAVTLGTSYD
BT1878          HPL------------KSDWEVYTKYGYFPTDLT--------K-----AESVSSTLESVYD
BT2199          HAEIA-------SVGRDGFEYYDKLGYVPYPE---------V-----PEATAKTLEYAYA
BT2629          HLRGT-------ASGRLGYESYNQLGYVANNIG--------I-----GQNVARTLEYAYN
BT3130          HPN-------------SPFEVWEKYGFMPENIQ--------------TQSVSITLEQAFD
BT3527          HPQVS-------STGRLGYDYYNKLGYVPYNVG--------I-----NENAARTLEYAYN
BT3773          GQN-----------G--VPYVMAKGYIPADKI--------------HEATSIAMEYAVD
BT3784          HLRGT-------ASGRLAYDAYNKLGYVPNNIG--------I-----GQNVARTLEYAYN
BT3858          LPWLRCPLTEL-------DKFYQEKGFFPALNPGEEETCKAVHSFERRQAVAVMLGNCYD
BT3962          HPRVS-------TTGRRGYEYYNKLGYVPYDVK--------I-----NENAARTLEYAYD
BT3963          YY-------------EGVADYMKLGYIPLDKS--------------GTAASSTLEYAYD
BT3965          NY-------------DCLPEYREMGYVPFDKE--------------AESVSKTLEYAYD
BT3990          HPEVS-------STGRLGYEYYNKLGYVPYDVK--------I-----NENAARTLEYAYD
BT3991          TTGIKCPDLVLPHLMPKAKYYKNAIGYIPCDRE--------------NESVAKALEYAYD
BT3994          DNY-------------RGIGLYKQLGYIPYNVT--------DHYNAENWSLSKTLEYAFD
BT4073          GN-------------------DSLGYTPE-----------------PLSISYTLEYAYA
BT4092          GPWGG-------ANGRGFWKEYFQLGYVPYPES--------M-----GS-SAQTMEYAYD
BT4093          GSKSQ------DVETRPGLKQYLDKGYYN---------------------ASIQLEYTSA
                                         *:                        :  :

BT1032          DFALSRFADALGKKKDA-EMFYKRSLGYKHYYSKEFGTFRPILPDGTFYSPFNPRQGEN-
BT1769          QWCLSRIAQALNKKEEA-EYYLKCSYNYRNLYNKETAFFHPKDKEGQWIEPFDYRFPGGM
BT1878          DYAAADMARRMGKEEDA-AYFAKRADYYKNLFDSQTNFMRPRKADGTWKSPFNPSDVGH-
BT2199          DWCIARFAESLGKQDIA-DQYYQKAPNYRNLYYPEHGFMWTKDAKGNWRDRFDATEWGG-
BT2629          DWAIYTLGKKLGKPENEIDIYKKHALNYKNVYHPERKLMVGKDNKGVFNPNFDAVDWSG-
BT3130          DWCVAQLAAKLNKDADY-QRFHKRSEYYRNLFHPKTKFFQSKNDKGEWIEPFDPYQYGG-
BT3527          DWCIYQLGKALNKPKKEIEIFAKRAMNYKNLYDPEHKLMRGKNEDGKFQSPFNPLKWGD-
BT3773          DWGIAAMAQKMGKTADY-ETFSKRAHYYKNYFDSSIHFIRPKLEDGSWRTPYDPARSI--
BT3784          DWTIYTLGKKLGKPASEIDIFKQRALNYKNVYHPKRKLMVGKDDKGVFNPKFDAVDWSG-
BT3858          NWCLAQIARTLNKTDDY-KKFMRMSYTYRNVYNAETGFFHPKNKDGKFIEPFDYRYSGGQ
BT3962          DWCIYRMGEKLGRPAEELELYKSRSQNYRNLFDPETKLMRGKNADGTFQTPFNPFKWGD-
BT3963          DWTIYQTALKAGNKEIA-DTYRKRALNYRNIYDTSIGFARPRYSDGTFKKEFDVLQTYGE
BT3965          DYCIAQAAKKLGKEDDY-HYFLNRALSYQTLIDPETKYMRGRDSKGDWRTPFTPVAYQGP
BT3990          DWCIYRLAKELKRPKKEISLFAKRAMNYKNLFDKESKLMRGRNEDGTFQSPFSPLKWGD-
BT3991          DWCISIFAEAMNDFENK-AKYERFAKAYEFYFDKSTRFMRGLDSNGEWRTPFNPRAST--
BT3994          DYCIAEMANKMGKKEIA-DEFYKRSQNYKNVYNPATSFMQPRDDKGNFIKDFKADEYTP-
BT4073          DWCVAQLAKALGKEEDA-KRFYEKGQAYRNMFDAEKGWFRPRNADGSWKAWPENA-----
BT4092          DFCGYQLAKMVGNKHYQ-EVFARQMYNYKNVFDKSIGFMRGKGVDGKWQEPFDPLEWGG-
BT4093          DFAIGQFALHAVGDEFASWRYFHFARSWKNLYNSDTGWLQSRNPDGSWKSL---------
                ::     .            :      :.               .* :

BT1032          --FEPNPGFHEGNSWNYTFYVPHDVYGLAKLMGGKKPFVNKLQMVFDEGL----------
BT1769          GA---REYYGENNGWVYRWDVPHNVADLISLMGGNEQFIANLDRTFTEPLGRSKY----A
BT1878          -AESTGGDYTEGNAWQYTWHVQHDVPGLIALFGGEEPFLNKLDSLFTVKL----------
BT2199          -------PFTEGSSWHWTSVFHDPEGLSELMGGHEPMIARLDSMFVAPNTYNYGTYGFV
BT2629          -------EFCEGNSWHWSFCVFHDPQGLINLMGGKKEFNAMMDSVFVISGKLGME-SRGM
BT3130          ---NGGHPFTEGNAWQYFWYVPHNIQALMELTGGTKAFEQKLDTFFTSTY-K-S------
BT3527          -------AFTEGNSWHYTWSVFHDPQGLIDLMGGKDGFNQMMDSVFILPPVFDDSYYGGV
BT3773          ---HGVGDFCEGNGWQYTFFAPQDPYGLISLFGGDKPFTSKLDSFFTNNDSM-G------
BT3784          -------EFCEGNSWHWSFCVFHDPQGLIDLMGGKKEFNNMMDSVFVIPGKQGME-SRGM
BT3858          GA---RGYYGENNGWIYRWDVQHNPADLIALMGGQASFIERLNQTFNEPLGRSKF----D
BT3962          -------AFTEGNSWHYTWSVFHDVQGLADLMGGRKMFVSMLDSVFNLPPIFDDSYYGGV
BT3963          GFI-------EGNSWNFSFHVPHDVFGMIDLMGGEKTFVQKLDELFSMHLPEKYY-----
BT3965          GSVHGWGDITEGFTMQYTWYVPQDVQGYINEA-GKELFRKRLDELFTVELPDDIP-----
BT3990          -------AFTEGNSWHYTWSVFHDPQGLIDLMGGKEMFVTMMDSVFAVPPIFDDSYYGQV
BT3991          ---HRSDDYCEGTAWQWTWFVPHDVEGLVKLMGGEDAFVEKLDSLFTVDSSLDG------
BT3994          -------HICESNGWQYFWSVQHDIDGLIDLTGGKNRFAEKLDSMFTYHPAA-D------
BT4073          -LTEEWYGCIESNAYQQGWFVPHDVPGMVELMGGKEEVIANLTNLFDHTPSDML------
BT4092          -------PFCEGNAWHYTWSVFHDVQGLIDLFGSDEKFTTKIDSVFTIPNIIKPGTYGGV
BT4093          -----GEDFRESTYKNYFWMVPYDIAGLVEIIGGKEKAEKRLDEFFTRLDAG--------
                          *.      : .  :         .       :   *
```

```
BT1032      --------------YDPANEPDIAYAHLFSYFKGEEWRTQKETQRLLDKYFTTKPDGIPG
BT1769      FYAKLPDHTGNVGQFSMANEPSLHVPYLYNY-AGQPWKTQKRIRQMLKTWFRNDLMGIPG
BT1878      -ETTQADVTGLIGQYAHGNEPSHHVTYLYAL-AGRPERTQELIREIFDTQYKNKPDGLCG
BT2199      IHEIAEMVALNMGQYAHGNQPVQHAIYLYDY-IGQPWKTQYHLRNVMDKLYNSGSKGYCG
BT2629      IHEMREMQVMNMGQYAHGNQPIQHMVYLYNY-SSEPWKAQYWIREIMNKLYTAGPDGYCG
BT3130      -EQMNHNASGFVGQYAHGNEPSHHVAYLYNF-AGQPWKTQKYVSHILNTLYNNTSSGYAG
BT3527      IHEIREMQIMNMGNYAHGNQPIQHMLYMYNY-SGQPWKAQHWIREVMDKLYTPAPDGYCG
BT3773      -EGASSDITGLIGQYAHGNEPSHHIAYLYTY-AGEQWKTAEKVRFIMSDFYTDQPDGIIG
BT3784      IHEMREMQVMNMGQYAHGNQPIQHMVYLYNY-SGEPWKAQHWVREIMDKLYTAGPDGYCG
BT3858      FYHQLPDHTGNVGQFSMANEPCLHIPYLYNY-AGQPWMTQKRIRVLLNQWFRNDLMGVPG
BT3962      IHEIREMEIANMGNYAHGNQPIQHMIYLYNY-AGEPWKAQYWLREVMNRLYFATPDGYCG
BT3963      EHNEDITEECLVGGYVHGNEPSHHVPYLYAW-TSQPWKTQYWLREILNKMYKNDINGLGG
BT3965      GAH---DIQGRIGAYWHGNEPCHHVAYLYNY-LKEPWKCQKWIRTIVDRFYGNTPDALSG
BT3990      IHEIREMTVMNMGNYAHGNQPIQHMIYLYDY-AGQPWKAQYWLRQVMDRMYTPGPDGYCG
BT3991      -ETTSADISGLIGQYAHGNEPSHHVIHLYNY-VNRPWRTQELVDSVYRSQYANNVDGLSG
BT3994      -EELPIFSTGMIGQYAHGNEPSHHVIYLFNA-VGQQNLTQKYVAKVMNELYKNEPAGLCG
BT4073      ----------WNDYYNHANEPVHFVPFLFNQ-LDVPWYTQKWTRYICKNAYANKVEGIVG
BT4092      IHEMKEMELANMGQYAHGNQPIQHMPYLYCY-AGQPWKTQYWVRQIVERLYNSTEKGYPG
BT4093      ---------YNDAWFASGNEPSFHIPWIYNW-IGRPYKTQEIINRVLNEQYSSKIDGLPG
                          :  .*:*      ::                :    :     .  *

BT1032      NDDTGTMSAWAIFNMIGFYPDCPGLPEYALTTPVFNKVTIRLDPKWYKENELVIESNRTG
BT1769      DEDGGGMTSFVVFSSLGFYPVTPGLPAYTIGSPLFTDAKIRL----SNGAVFEIEAKNAS
BT1878      NDDCGQMSAWYMFSAMGFYPVDPVSGDYVFGAPQLPKIVLHL----ADGKTFTVIAENLS
BT2199      DEDNGQTSAWYVFSAMGFYPVCPGMPEYAIGSPLFKKVTLHL----PEGKNFVVSAADNA
BT2629      DEDNGQTSAWYVFSALGFYPVCPGTDEYIIGTPLFKSAKLHL----ENGKTITIKADNNQ
BT3130      NDDCGQMSAWYVFSAMGFYPVNPADGRYIIGSPLLDECTLKL----AGNKEFRIRTIRKS
BT3527      DEDNGQTSAWYVFSAMGFYPVCPGTDEYILGTPYFKQMKLHL----ENGKTVTISAPNNG
BT3773      NEDCGQMSAWYLLSAMGFYQVNPSDGVFAFGSPRFKKIEVKV----RGGKVFTVEAPNNS
BT3784      DEDNGQTSAWYVFSALGFYPVCPGTDQYILGTPLFKSAKLHL----ENGKTVTIKASNNN
BT3858      DEDGGGMTAFVVFSMMGFYPVTPGSPTYNIGSPVFQSAKMEV----GDGHYFEIIAENYA
BT3962      DEDNGQTSAWYVFTALGFYPVCPGSNEYVMGAPYFKKATITL----ENGKKLEISAPKNS
BT3963      NDDCGQMSAWYLFSVMGFYPVCPGTDQYVLGAPYLPYLKLTL----PNGKTLEIKAPGVS
BT3965      NDDCGQMSAWYMFNCIGFYPVAPSSNIYNIGSPCAEAITVRM----SNGKNIEMTADNWS
BT3990      DEDNGQTSAWYVFSALGFYPVCPGTDEYVMGTPLFKKATLHF----ENGNSLVIDAPNNS
BT3991      NEDCGQMSAWYILNSMGFYQVCPGKPVYSIGRPAFDKAVINL----PGEKTFSIVAKNNS
BT3994      NEDCGQMSAWYVFSAMGFYPVNPVSGKYEIGTPLFPEMKLHL----ANGKTFTVLAPKVS
BT4073      NEDVGQMSAWYILAASGIHPSCPGNTRMEITSPVFDKVEFNLDSKYHQGKVFTIIAHNNN
BT4092      DEDQGGMSSWYILSSLGIYAVCPGTDEYVIGSPLFKKATITM----ENGNKFVIEAPENS
BT4093      NDDLGTMGAWYVFACIGLYPEIPGVGGFTVNTPIFSSVKVHL----KKG---DIVIKGGS
            ::* *   :: ::   *::   *      .  *      . .           :

BT1032      SETLYINKVLLDGKKFNKYRITHDELVHGKRLIFDLK-----------------------
BT1769      TDNKYIQSATLNGKEWNKSWFSHDDLMSGGKLVLVMGNKPNKTWASGAEDVPPSLEIK--
BT1878      KEHKYVDSITLNGEPYTKNTISHEDILKGGTLVYKMK-----------------------
BT2199      ADRPYIRKALLNGQEFTRNYLTHDELKQGGELNLSMDSVPNQQRGTQPADFPYSYSK---
BT2629      LDNRYIKEMKVNGKSQTRNFLTHDQLIKGANIQFQMSPVPNKQRGTTEKDVPYSLSFE--
BT3130      PEDIYIQSVTLNGKKHKDFFITHQDIMNGGTMVFKMGKKPSGWGK---------------
BT3527      DDKRYISSMTLNGKDHTKNYVTHEDLMNGASITFKMDSKPNEQRGTKETDFPYSFSNEFK
BT3773      KDNIYIQKVYLNGKPYHKSYITYDDIINGSTLKFEMGKKPAKNFGKASANRPIVLNK---
BT3784      TDNRYVKDMKVNGKAFTRNYLTHDQLLKGANIQYQMSPTPNKQRGTTEKDIPYSLSFE--
BT3858      PDHKYIQSATLNGTPWNKPWFSHADIQNGGRLVLQMGDKPNKKWGIASDAVPPSSESLPE
BT3962      DANRYIRSLNYNGKNYTKNYLNHFDLLKGGRLVFDMDNKPNKGRGINESDFPYSFSRDNK
BT3963      DKKRYVQSLKLNGESYDKMYITHEDILKGGVLEFKMSASPNKRRGVSVQDKPYSLTNGIN
BT3965      PKNLYVKELYVNGKKYDKSYLTYDDIRDGVKLRFVMSGKPNYKRAVSDEAVPPSISLPEK
BT3990      TENFYIDSMSFNGADHTKNYLRHEDLFKGGTIKVDMSNRPNLNRGTKEEDMPYSFSKE--
BT3991      KSNKYIESVLLNGKPLDTPFFGHQDIVAGGVMEIRMTDHPTQWGVRQ-------------
BT3994      KENIYIQSIKVDGQPYNKTYLTHEQIMSGTTVEFEMGNTPLVEVEFEEQTQ---------
BT4073      TNNLYIQKALLNGKEYNKCYLDFAEIAAGGTLELFMGDKPNTEWGVLSNI----------
BT4092      KENLYIQSATLNGRLLDKNYIHYDDIAEGGVLKFEMGSQPNKERCTSKYAAPFSLSKE--
BT4093      EKDIYIKSMKLNGKSYESTWIDWDQLNSGATIEYRTSGKPDMKWGAK--VVPPSFP----
                *: .   :*       .   ::  *  :

BT1032      ----------
BT1769      ----------
BT1878      ----------
BT2199      ----------
BT2629      ----------
BT3130      ----------
BT3527      KKK-------
BT3773      ----------
BT3784      ----------
BT3858      ----------
BT3962      ----------
BT3963      ----------
BT3965      TMKYKSSIGF
BT3990      ----------
BT3991      ----------
BT3994      ----------
BT4073      ----------
BT4092      ----------
BT4093      ----------
```

Figure S3 Expanded *Bt* GH92 sequence alignment used to map sequence conservation in Figure S1.
